# Signaling through the dystrophin glycoprotein complex affects the stress-dependent transcriptome in *Drosophila*

**DOI:** 10.1242/dmm.049862

**Published:** 2023-01-31

**Authors:** Travis D. Carney, Rucha Y. Hebalkar, Evgeniia Edeleva, Ibrahim Ömer Çiçek, Halyna R. Shcherbata

**Affiliations:** ^1^ Hannover Medical School, Research Group Gene Expression and Signaling, Institute of Cell Biochemistry, Hannover 30625, Germany; ^2^ Mount Desert Island Biological Laboratory, Bar Harbor, ME 04609, USA; ^3^ xFOREST Therapeutics, Kyoto 602-0841, Japan; ^4^ Illumina Cambridge Ltd, Great Abington, Cambridgeshire CB21 6DF, UK

**Keywords:** Muscular dystrophy, Dystrophin glycoprotein complex, Dystroglycan, Stress response, *Drosophila*

## Abstract

Deficiencies in the human dystrophin glycoprotein complex (DGC), which links the extracellular matrix with the intracellular cytoskeleton, cause muscular dystrophies, a group of incurable disorders associated with heterogeneous muscle, brain and eye abnormalities. Stresses such as nutrient deprivation and aging cause muscle wasting, which can be exacerbated by reduced levels of the DGC in membranes, the integrity of which is vital for muscle health and function. Moreover, the DGC operates in multiple signaling pathways, demonstrating an important function in gene expression regulation. To advance disease diagnostics and treatment strategies, we strive to understand the genetic pathways that are perturbed by DGC mutations. Here, we utilized a *Drosophila* model to investigate the transcriptomic changes in mutants of four DGC components under temperature and metabolic stress. We identified DGC-dependent genes, stress-dependent genes and genes dependent on the DGC for a proper stress response, confirming a novel function of the DGC in stress-response signaling. This perspective yields new insights into the etiology of muscular dystrophy symptoms, possible treatment directions and a better understanding of DGC signaling and regulation under normal and stress conditions.

## Teaser

**Summary:** The muscular dystrophy-associated dystrophin glycoprotein complex (DGC) functions as an integral component of multiple signaling pathways and is involved in stress-dependent regulation of gene expression.

## INTRODUCTION

**Fig. 1.**
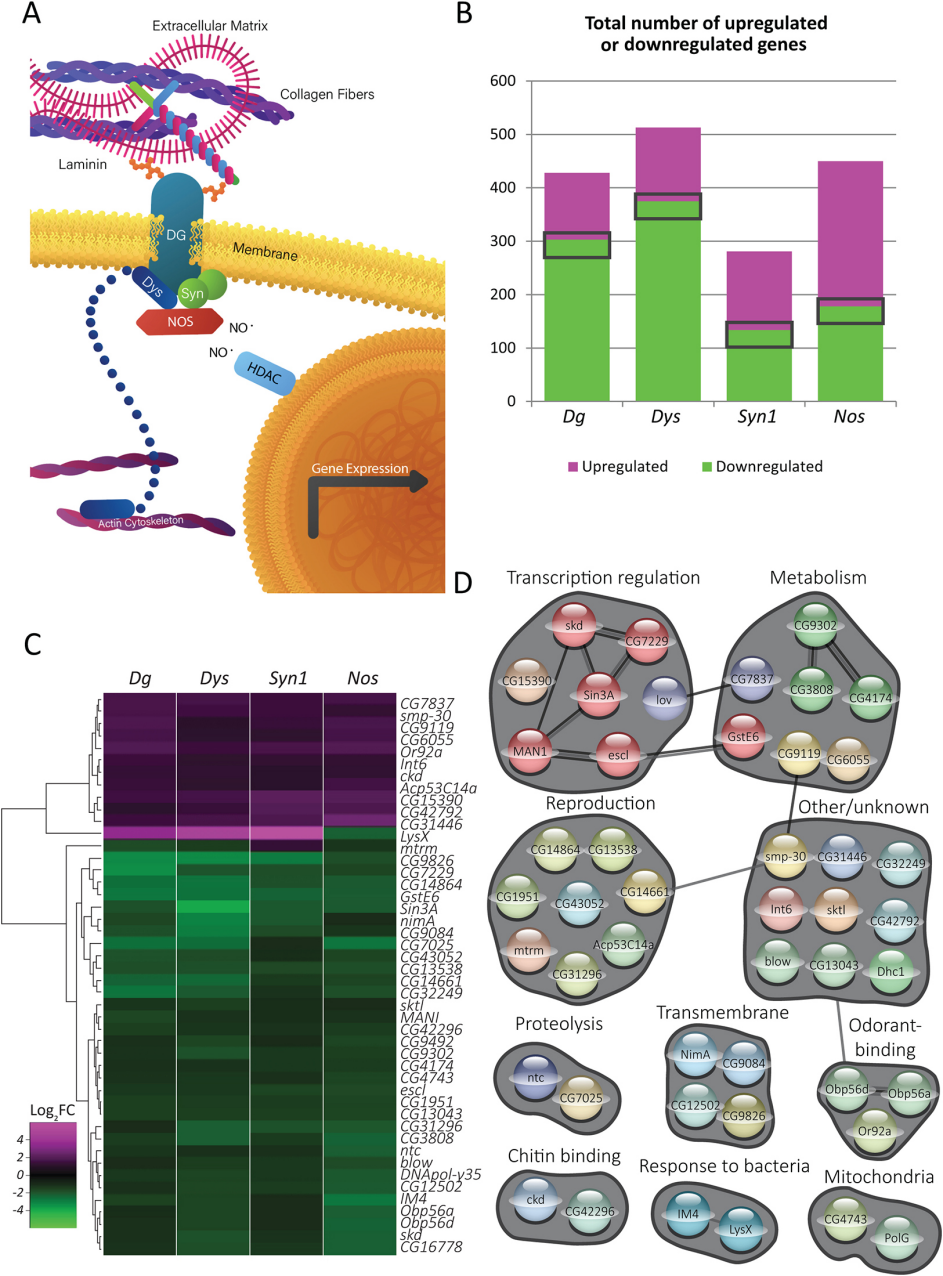
**DGC-dependent signaling exerts a defined transcriptional response.** (A) Schematic of the DGC and associated proteins. The transmembrane protein Dystroglycan (Dg) is the core component of the complex. Its glycosylated extracellular domain associates with the extracellular matrix, whereas its intracellular portion associates with Dystrophin (Dys) and Syntrophin (Syn), which can act as structural and signaling scaffolds, attaching to the intracellular cytoskeleton as well as to signaling molecules such as nitric oxide synthase (Nos). The nitric oxide (NO) produced by Dys-associated Nos can nitrosylate intracellular proteins such as histone deacetylases (HDACs), affecting the expression of downstream genes. (B) Numbers of genes found to be dysregulated in flies mutant for each of four DGC components: *Dg* (*DgO55/DgO86*), *Dys* [*Df(3R)Exel6184*], *Syn1* [*ΔSyn15-2/Df(3L)BSC450C*] and *Nos* [*ΔNos^15^/Df(2L)BSC230*]*.* The numbers of dysregulated genes ranged from 281 for *Syn1* to 513 for *Dys*. The gray rectangles represent the 46 genes that are dysregulated in all four mutants. (C) Heat map illustrating the 46 genes that are dysregulated in all four mutants assayed. Eleven genes are upregulated and 33 downregulated in all mutants; only two genes, *LysX* and *mtrm*, are differentially regulated depending on the genotype. (D) STRING-based clustering of the gene products of the 46 genes that are dysregulated in all four DGC mutants. The clusters are labeled according to the functions and processes in which the genes are involved. Gray lines indicate physical interactions, both putative and experimentally determined; different shades of gray are used for contrast purposes.

The dystrophin glycoprotein complex (DGC) is a cell membrane-associated protein complex that connects the extracellular matrix (ECM) to the cytoskeleton of the cell. The core components of the DGC are the transmembrane, ECM-associated protein dystroglycan (Dg in *Drosophila*), cytoplasmic dystrophin (Dys in *Drosophila*) and cytoplasmic syntrophin (Syn1 in *Drosophila*) proteins (Fig. 1A). DGC dysfunction is associated with a group of diseases, the muscular dystrophies (MDs), which have deleterious and sometimes fatal effects on muscles and the nervous system. For example, loss of dystrophin results in Duchenne MD (Ervasti and Campbell, 1993) and aberrant glycosylation of dystroglycan leads to severe forms of congenital and late-onset MDs (Barresi et al., 2004; Bigotti and Brancaccio, 2021; Brun et al., 2018; Goddeeris et al., 2013; Imae et al., 2021; Joseph and Campbell, 2021; Mantuano et al., 2021; Munot et al., 2022; Xie et al., 2022; Yatsenko et al., 2021). In contractile muscle cells, DGC-dependent linkage of the ECM and intracellular cytoskeleton accounts for the stability and mechanical stress resistance of the sarcolemma, limiting contraction-initiated damage (Demontis et al., 2013; Gao and McNally, 2015; Larsson et al., 2019). MD patients experience progressive muscle degeneration and often die because of heart or respiratory failure. Duchenne MD patients also suffer from cognitive impairment (Perronnet and Vaillend, 2010), and some patients with congenital MDs exhibit structural brain abnormalities, intellectual disabilities, abnormal neuronal migration and changes in white matter (Cohn, 2005). In addition to muscular and neurological deficits, it has been reported that DGC deficits can result in reduced male fertility in mice (Chen et al., 2017; Hernandez-Gonzalez et al., 2005), and in *Drosophila*, these deficits cause systemic problems such as an inability to maintain temperature homeostasis (Takeuchi et al., 2009).

The DGC is also involved in cell signaling. Syntrophins have multiple protein-protein interaction motifs and can serve as adaptor proteins capable of binding to heterotrimeric G proteins and neuronal nitric oxide synthase (Nos in *Drosophila*), among other signaling modules (Cacchiarelli et al., 2010; Xiong et al., 2009; Zhou et al., 2006). Syntrophins constitute a cytoplasmic platform to which neuronal Nos can bind and produce nitric oxide (NO), an important signaling molecule that acts via the nitrosylation of intracellular proteins. This nitrosylation serves to inhibit mammalian histone deacetylase 2 (HDAC2), leading to the activation of HDAC2-responsive genes, including microRNAs necessary for the differentiation of muscle progenitor cells (Cacchiarelli et al., 2010). In flies, Dg and Nos signal cooperatively via a feedback loop in which Nos activity promotes the expression of a cluster of microRNAs that directly target the *Dg* transcript. Consistent with a functional DGC providing a signaling platform for Nos, overexpression of Dg results in increased production of NO by Nos (Yatsenko et al., 2014b). As the DGC is a mechanosensory complex, this conserved link demonstrates an important connection between intercellular forces and transcriptional activity.

Another important example of the link between the DGC, signaling and downstream transcriptional activity is demonstrated by the relationship between the DGC and Hippo signaling in both mice and *Drosophila*. In murine cardiomyocytes, the phosphorylated effector of Hippo signaling, Yap, is directly bound by the Dg ortholog Dag1, and thus sequestered by the membrane-associated complex. This sequestration serves as an important control for cardiomyocyte proliferation, evidenced by an overproliferation phenotype at the site of cardiac injury in Hippo-Dag1 double mutants (Morikawa et al., 2017; Vita et al., 2018). Similarly in flies, the Hippo effector Yorkie (Yki) physically associates with the DGC, as does another component of the signaling cascade, Kibra (Kbr). These interactions were shown to promote the maintenance of muscle integrity during aging in adult flies (Yatsenko et al., 2020).

A fundamental factor affecting organismal physiology is temperature. Genomics studies in various model organisms, such as mice, flies, worms and yeast have demonstrated that in response to heat stress, a rapid and transient reprioritization of the gene expression program occurs. These changes include repression of genes involved in growth and cell proliferation, rearrangement of DNA and chromatin, regulation of energy metabolism and the redox state of the cells, alternative splicing and proteostasis (Evans, 2015; Gasch et al., 2000; Lopez-Maury et al., 2008; Sorensen et al., 2016). Exposure to high ambient temperatures can result in high morbidity and mortality (Shindell et al., 2020; Vicedo-Cabrera et al., 2021). Extreme or prolonged heat can overwhelm thermoregulatory capacity even in healthy persons, but it is especially dangerous for patients with muscle disorders (Cheshire, 2016). For example, patients with MDs have a high risk of malignant hyperthermia and heart failure as a response to anesthetic agents (Hayes et al., 2008; Ohkoshi et al., 1995; Rohde et al., 2014). In these individuals, a drastic increase of Ca^2+^ in skeletal muscle leads to sustained contractions, heat generation and a dangerous increase in body temperature. In *Drosophila* larvae, *Dg* mutation also causes increased intracellular Ca^2+^ concentration and concomitant oxidative metabolism, resulting in a cold-seeking behavior that is rescued by the transgenic re-introduction of *Dg* (Takeuchi, et al., 2009). However, the role of the DGC in thermoregulation remains elusive. Moreover, as increasing worldwide environmental temperatures have dire health effects, particularly among urban dwellers and people with metabolic and cardiopulmonary disorders (Burkart et al., 2021; Ebi et al., 2021; Shindell et al., 2020; Vicedo-Cabrera et al., 2021), it is important to understand the influence of heightened temperature not only on patients with MDs but also on the wellbeing of healthy individuals.

In addition to genetic disorders (such as MDs), other physiological and pathological stimuli (e.g. fasting and cachexia) can cause muscle wasting. Starvation usually results in muscle atrophy, which is loss of muscle mass due to an increase in protein degradation or a decrease in protein synthesis (Piccirillo et al., 2014). Muscle loss is an integral feature of systemic diseases including cancer, cachexia, cardiac failure, AIDS and sepsis. One important aspect of the stress response to dietary restrictions is an alteration in muscle metabolism that leads to the decreased usage of carbohydrates, so that they can be spared for the organs and tissues in which glucose is essential, such as the central nervous system. Loss of muscle mass due to aging, also known as sarcopenia, is often associated with muscle disuse, fasting, extrinsic changes in innervation, stem cell function and endocrine regulation of muscle homeostasis (Demontis et al., 2013). This loss of muscle mass is triggered, in part, by the reduced content of the DGC in membranes, the integrity of which is vital for muscle health and function. In fact, the reduction in DGC content appears to precede and promote age-associated muscle atrophy and associated weakness and frailty (Bengtsson et al., 2022). Conversely, stabilization of the DGC on the muscle membrane markedly attenuates atrophy (Eid Mutlak et al., 2020). Therefore, understanding the role of the DGC in muscle maintenance upon metabolic stress is extremely important for understanding the molecular mechanisms that cause muscle atrophy during aging and in various catabolic states (e.g. starvation, type 2 diabetes). However, it is not clear how this mechanosignaling complex affects various components of the cellular stress response.

The components of the DGC are evolutionarily conserved from *Drosophila* to mammals but exist in flies with significantly less redundancy (Greener and Roberts, 2000). As in mammals, the DGC components in *Drosophila* are expressed not only in muscles, but also in nervous and other tissues (Bogdanik et al., 2008; Dekkers et al., 2004; Marrone et al., 2011a; van der Plas et al., 2006). Flies deficient for Dys or Dg develop phenotypes similar to those seen in MD patients, both in the muscle and the nervous systems. They experience a shortened lifespan, decreased mobility, age-dependent muscle degeneration and defective neuron differentiation (Shcherbata et al., 2007). Using *Drosophila melanogaster* as an MD model, our laboratory previously demonstrated that temperature, metabolic stress, oxidative stress and aging can enhance muscle degeneration in flies mutant for *Dys* or *Dg*, and can promote degeneration even in wild-type flies (Kucherenko et al., 2011). In addition, we found a group of both dystrophy- and stress-dependent microRNAs that are upregulated or downregulated due to high-temperature stress in wild-type flies but fail to change their expression levels under high-temperature stress in dystrophic flies (Marrone et al., 2012). These data support a concept that there is a signaling pathway under stress between the cell membrane-associated DGC and nuclear gene expression.

To find novel DGC-dependent genetic pathways and to further investigate the relationship between the DGC and the transcriptional stress response, we performed RNA-sequencing (RNA-seq) analysis from whole flies under unstressed conditions, under temperature stress and under metabolic stress. We compared control flies to mutants of four different DGC components: Dg, Dys, Syn1 and Nos (Table S1). From this analysis, we uncovered a group of genes that are dysregulated in all four DGC mutants in unstressed conditions, representing genes that are specifically regulated by DGC-dependent signaling mechanisms. Consistent with the DGC being a coherent signaling center, nearly all of these genes were similarly upregulated or downregulated in all four mutants. By comparing stressed to unstressed control flies, we identified several hundred genes that are differentially regulated in response to either temperature or metabolic stress. Consistent with previous studies (Lecheta et al., 2020; Sorensen et al., 2005; Telonis-Scott et al., 2013; Zhou et al., 2012), we found that the majority of the dysregulated genes are downregulated by temperature stress, but more genes are upregulated by dietary restriction. This pattern is also evident in aged flies and those subjected to oxidative stress (Landis et al., 2004). Strikingly, only a small minority of genes are commonly dysregulated by both stresses, illustrating the distinct transcriptional responses to temperature and metabolic stress. Finally, we identified sets of genes for which differential expression patterns under stress are perturbed in mutants with a non-functional DGC, constituting groups of genes dependent on the DGC signaling pathway for a proper stress response. A whole-genome overview of organismal transcriptional changes is an important descriptive analysis of the DGC-dependent stress response. This work reveals a novel function of the DGC in stress-response signaling. The view of the DGC as a regulatory unit involved in the stress response will give new insights into the etiology of MD symptoms and possible directions of symptomatic treatment and relief.

## RESULTS AND DISCUSSION

### DGC-dependent signaling exerts a defined and consistent transcriptional response

To validate that the DGC complex exerts a coherent transcriptional effect, we first investigated the effects of multiple DGC mutants – *Dg*, *Dys*, *Syn1*, and *Nos* – in comparison to control flies (Table S1). By using these four different DGC mutant alleles and by hierarchical clustering of dysregulated genes, we were able to distinguish between genes downstream of the DGC complex as a whole and those that could be affected by individual mutant lesions owing to functions or pleiotropic effects unrelated to the DGC. By this rationale, if the DGC complex is responsible for distinct and regulated transcriptional outputs, we would expect to see that downstream genes are dysregulated similarly in each of the mutants. If, on the contrary, downstream genes were to exhibit differential dysregulation in each of the mutants, this would indicate that these transcriptional perturbations are not a result of the combined effect of the loss of DGC complex function, but rather result from unrelated or pleiotropic effects.

First, we filtered for genes that are upregulated or downregulated at least twofold in any of the DGC mutants compared with wild type, irrespective of stress (Fig. 1B). Each of the four DGC mutants exhibited a different number of total dysregulated genes, from 281 in *Syn1* mutants to 513 in *Dys* mutants. The majority of these genes were distinct to each genotype, implying that these were genetic lesion-specific effects. For this reason, we chose to focus instead on the subset of genes that were commonly dysregulated in all four mutant genotypes. With these criteria, we found that 46 genes were dysregulated by at least twofold in all mutant genotypes (dark rectangles in Fig. 1B,C; Table S2). Strikingly, 96% (44 of 46 genes) of these genes exhibited a matching upregulation or downregulation pattern in all DGC mutants. This strongly suggests that the DGC is integral to a defined transcriptional program. Therefore, we conclude that these genes likely represent ‘DGC-dependent’ genes.

As indicated above, each individual DGC lesion resulted in hundreds of dysregulated genes, whereas far fewer (46 genes) were commonly dysregulated in all four mutants – the most stringent criterion applied here. Applying the less stringent requirement that genes were dysregulated in only three out of the four mutants, an intermediate number of genes was the result. For example, if we disregarded the *Nos* loss-of-function (LOF) mutant and looked at only genes that were dysregulated in mutants of the core DGC components *Dg*, *Dys* and *Syn1*, we found that an additional 25 genes were dysregulated, or over 50% more genes (Table S3, genes shown in bold). We believe that the core DGC components Dg, Dys and Syn1 might behave more similarly to one another transcriptionally owing to cellular perturbations that result from a physically defective DGC. Mutation of *Nos*, in contrast, is well known to result in transcriptional changes due to nitrosylation of histone deactylases, as previously mentioned. Therefore, it was important that we required that genes be dysregulated in the *Nos* mutant as well as in the *Dg*, *Dys* and *Syn1* mutants, thereby actively selecting for genes that exhibited a transcriptional effect directly downstream of the DGC and increasing our confidence that they can truly be considered as DGC-dependent genes (Fig. 1B-D).

*LysX* and *mtrm* were differentially regulated across the four mutants*. mtrm* was downregulated in all mutants except *Syn1*, in which it was slightly upregulated; the expression changes were much more modest than those exhibited by *LysX*. The expression of *mtrm* is highly female biased and it encodes a protein required for proper chromosome segregation during meiosis (Harris et al., 2003). As we used male flies for the sequencing experiments, *mtrm* expression level was low in most samples (e.g. *mtrm* was among the lowest-expressing 25% of transcripts detected in *Canton-S* controls and the bottom 5% of *Dg* mutant transcripts).

*LysX* was the strongest upregulated gene in *Dg*, *Dys* and *Syn1*, but it was downregulated in the *Nos* mutant (Fig. 1C). This might indicate that *LysX* is regulated by the DGC complex and Nos via independent mechanisms. *LysX* is one of seven lysozyme genes in *D. melanogaster*, all of which are clustered on chromosome 2 near 61F. Lysozymes hydrolyze peptidoglycan in bacterial cell walls and, in other insects, they are often found in the hemolymph and participate in antimicrobial defense. In *D. melanogaster*, however, rather than immune defense, lysozymes are strongly expressed in the digestive tract and are believed to be involved in the digestion of bacteria in the food (Daffre et al., 1994). Interestingly, the ingestion of bacteria results in the induction of *Nos* in the digestive tract (Foley and O'Farrell, 2003), raising the possibility that the subsequent production of NO induces the expression of *LysX* and other lysozyme genes. Thus, in *Nos* mutant animals, *LysX* was downregulated, as we saw here (Fig. 1C). It remains unknown, however, why *LysX* expression was upregulated in the *Dg*, *Dys* and *Syn1* mutants. Interestingly, in the analysis mentioned above in which we filtered for genes dysregulated in the *Dg*, *Dys* and *Syn1* mutant genotypes (excluding the *Nos* LOF mutant), two more lysozyme genes emerged, *LysE* and *LysS*, both of which were strongly upregulated in DGC mutants (Table S3). This result indicates that the DGC actively inhibits the expression of these lysosome genes by a Nos-independent mechanism, and it is consistent with our hypothesis that Nos is required for their upregulation in the gut.

To attain a clearer picture of the types of proteins encoded by the DGC-dependent genes, we clustered the gene products by annotated function and physical interactions using the STRING database (https://string-db.org; Szklarczyk et al., 2021). Direct and indirect physical interactions are shown as lines between nodes (genes), which we have assembled manually according to their annotated biological processes (Fig. 1D). The products of the genes that are dysregulated in all four DGC mutants are quite broad in function and associated biological process, suggesting that DGC deficiency has wide-ranging effects on the organism. Despite this, several distinct clusters emerged from our analysis, including ‘reproduction’, ‘transcription regulation’ and ‘metabolism’. The finding that genes involved in reproduction are dysregulated by DGC mutations is consistent with the male infertility exhibited by dystrophin-utrophin double mutant mice (Chen et al., 2017; Hernandez-Gonzalez et al., 2005).

### Temperature and metabolic stress result in distinct transcriptional changes

**Fig. 2.**
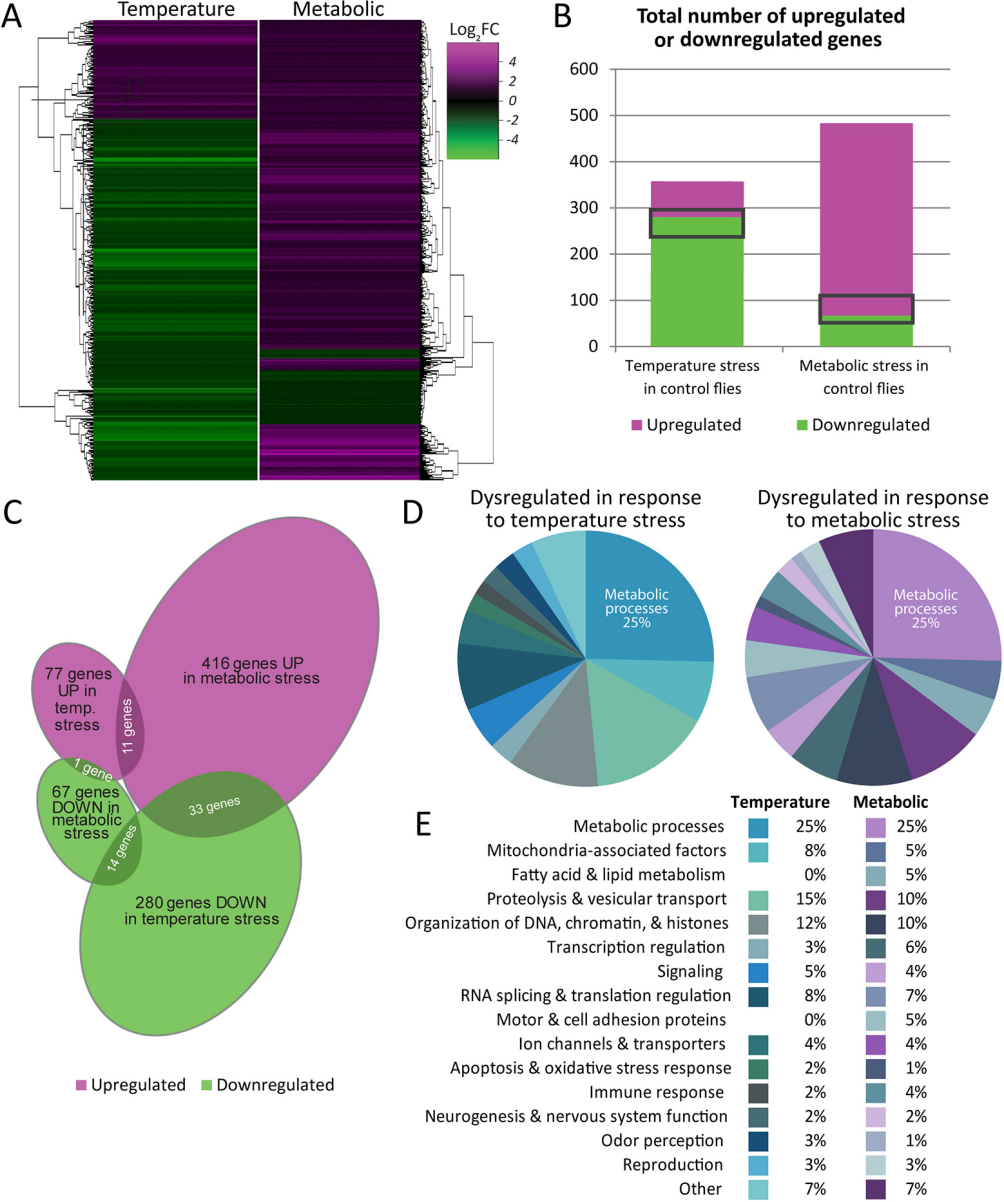
**Temperature and metabolic stress result in distinct transcriptional changes.** (A) Heat maps depicting all genes dysregulated in control flies by either temperature or metabolic stress, irrespective of their levels of expression in the DGC mutants. (B) The numbers of genes dysregulated in control flies by temperature stress (357 genes) or metabolic stress (483 genes). Temperature stress is much more likely to cause the downregulation of genes, whereas metabolic stress causes more upregulation. Gray rectangles indicate the 59 genes that were dysregulated by both stressors. (C) Venn diagram illustrating the upregulation (UP) and downregulation (DOWN) of genes as a result of temperature or metabolic stress. The overlapping regions add up to the 59 genes dysregulated by both stressors. (D,E) Categories of all genes with known functions that are dysregulated in response to temperature or metabolic stress. Pie charts (D) provide a visual depiction of the relative fraction each gene category constitutes of the total. The table (E) lists categories and percentages for both stress conditions. The largest category in both pie charts is ‘Metabolic processes’ (25%), and the categories in the pie chart extend in a clockwise direction in the same order as in the table. See also Fig. S2.

To investigate the genome-wide transcriptional effects of different stresses on adult flies, we determined the transcriptome-wide changes in gene expression of at least twofold in wild-type flies subjected to either temperature stress (33°C for 5 days) or metabolic stress (4 days with yeast paste-only diet; see Materials and Methods). These conditions resulted in very different transcription profiles (Fig. 2A,B). Temperature stress resulted in many more downregulated than upregulated genes (280 of 357 dysregulated genes were downregulated; 78.4%) (Table S4). This is similar to the results of a recent study that analyzed the human stress response to passive exposure to environmental heat at the transcriptomic level (Bouchama et al., 2017). This study revealed that the heat-reprogrammed transcriptome was predominantly inhibitory and that the differentially expressed genes encoded proteins that function in stress-associated pathways such as proteostasis, energy metabolism, cell growth and proliferation, and cell death and survival. The transcriptomic changes also included mitochondrial dysfunction, altered protein synthesis and reduced expression of genes related to immune function (Bouchama et al., 2017).

In contrast to temperature stress, metabolic stress is much more likely to cause upregulation (416 of 483 of dysregulated genes; 86.1%) (Fig. 2A,B; Table S5). The disparity we show between these different stress conditions is demonstrative of the fact that temperature and metabolic stress cause very different transcriptional outcomes. Heat shock protein (HSP) genes illustrate an important exception to this observation. Indeed, we found that of the 19 HSP genes we detected, most were weakly upregulated by temperature and downregulated by metabolic stress (Table S1). Unexpectedly, however, none of these genes was upregulated more than twofold [log_2_(fold change or FC)>1] by temperature. We propose that this weak upregulation is due to the relatively mild and chronic heat treatment to which we subjected our flies. Sixteen out of 19 HSP genes were weakly upregulated by temperature with an average fold change of 1.3, whereas two genes were weakly downregulated. The final HSP gene, *Hsp23*, was the only HSP gene to show a dysregulation of more than twofold and exhibited a downregulation in temperature-stressed control flies (this downregulation was also exhibited by DGC mutants, placing *Hsp23* as a ‘DGC-independent’ temperature stress-responsive gene; see below). Interestingly, a recent study demonstrated that even though *Hsp23* was upregulated by heat, its loss of function increased the tolerance of fruit flies to heat stress (Gu et al., 2021). It is possible that the intensity or duration of heat stress might affect the upregulation or downregulation of *Hsp23*; therefore, the temperature-responsive downregulation that we observed here might constitute part of a stress context-dependent survival mechanism.

Overall, a combined total of 840 genes were differentially expressed upon temperature or metabolic stress. Interestingly, the genes dysregulated in these two conditions exhibited little overlap: only 59 genes (7%) were differentially expressed in both conditions (Fig. 2B, gray rectangles; Table S6). Of these, 25 genes were similarly regulated in both stress conditions. The other 34 genes were differentially regulated, all but one being downregulated by temperature stress and upregulated in the metabolic stress condition (Fig. 2C; Table S6). As temperature and metabolic stress caused such disparate effects, it is possible that the 59 commonly dysregulated genes represent a general set of non-specific stress-responsive genes.

Next, we wanted to examine globally the types of genes that were dysregulated either by temperature stress or by metabolic stress. We analyzed the list of dysregulated genes in each stress condition (Fig. 2A) and binned them according to their annotated functions. As 93% of these genes were dysregulated by only one stress condition but not the other, we expected to see that very different types and classes of genes would appear in each list. Surprisingly, we found that the contrary was true. Not only were similar classes of genes dysregulated by both stresses, the proportion assigned to each class was remarkably similar in both (Fig. 2D). For example, of all the dysregulated genes with annotated functions, metabolic genes comprised 25% of both the temperature and metabolic groups (Fig. 2D,E). Similarly represented with very similar frequency in both temperature and metabolic stress conditions were genes such as those encoding mitochondria-associated factors (8% in temperature stress; 5% in metabolic stress), those related to proteolysis (15% in temperature stress; 10% in metabolic stress) and transcription regulation (3% in temperature stress; 6% in metabolic stress), as well as numerous other categories (Fig. 2D,E).

Our finding that the processes most affected in response to high temperature were genes involved in metabolic regulation is consistent with previous studies. It has been shown that thermal stress affects metabolic and physiological functions and depletes energy reserves in *Drosophila* (Klepsatel et al., 2016, 2019). Interestingly, a recent study demonstrated that the metabolism in stressed *Drosophila* depends on aerobic glycolysis – also known as the Warburg effect – rather than mitochondrial oxidative phosphorylation (Lee et al., 2015). Various genes involved in the electron transport chain and in ATP production were repressed, indicating poor mitochondrial fitness and suggesting that upon heat stress, there may be a switch in metabolism type in *Drosophila*.

Reproduction-related genes constitute 3% of genes dysregulated by temperature stress. It is not unexpected that reproduction is affected by thermal stress, as it has been reported that environmental stressors induce changes in endocrine state, leading to energy re-allocation from reproduction to survival (Meiselman et al., 2018; Ojima et al., 2018; Zwoinska et al., 2020). Stress-induced reproductive arrest has been documented not only in *Drosophila*, but also in other animals and humans (Boni, 2019).

Under metabolic stress, the most highly represented category of dysregulated genes was ‘metabolic processes.’ This group of genes was enriched for genes involved in aromatic amino acid metabolism and carbohydrate metabolism. The aromatic amino acids phenylalanine, tryptophan, and tyrosine are essential amino acids and are obtained in the diet, thus, the dysregulation of these genes and those involved in carbohydrate metabolism is consistent with a nutrient-deprivation state. We conclude that in response to stress, there is a common and reproducible set of processes that must be altered at the transcriptional level; although the specific genes that are dysregulated are stress-specific, the biological processes that are affected are common to different stresses.

### The transcriptional response to stress is defective in DGC mutants

**Fig. 3.**
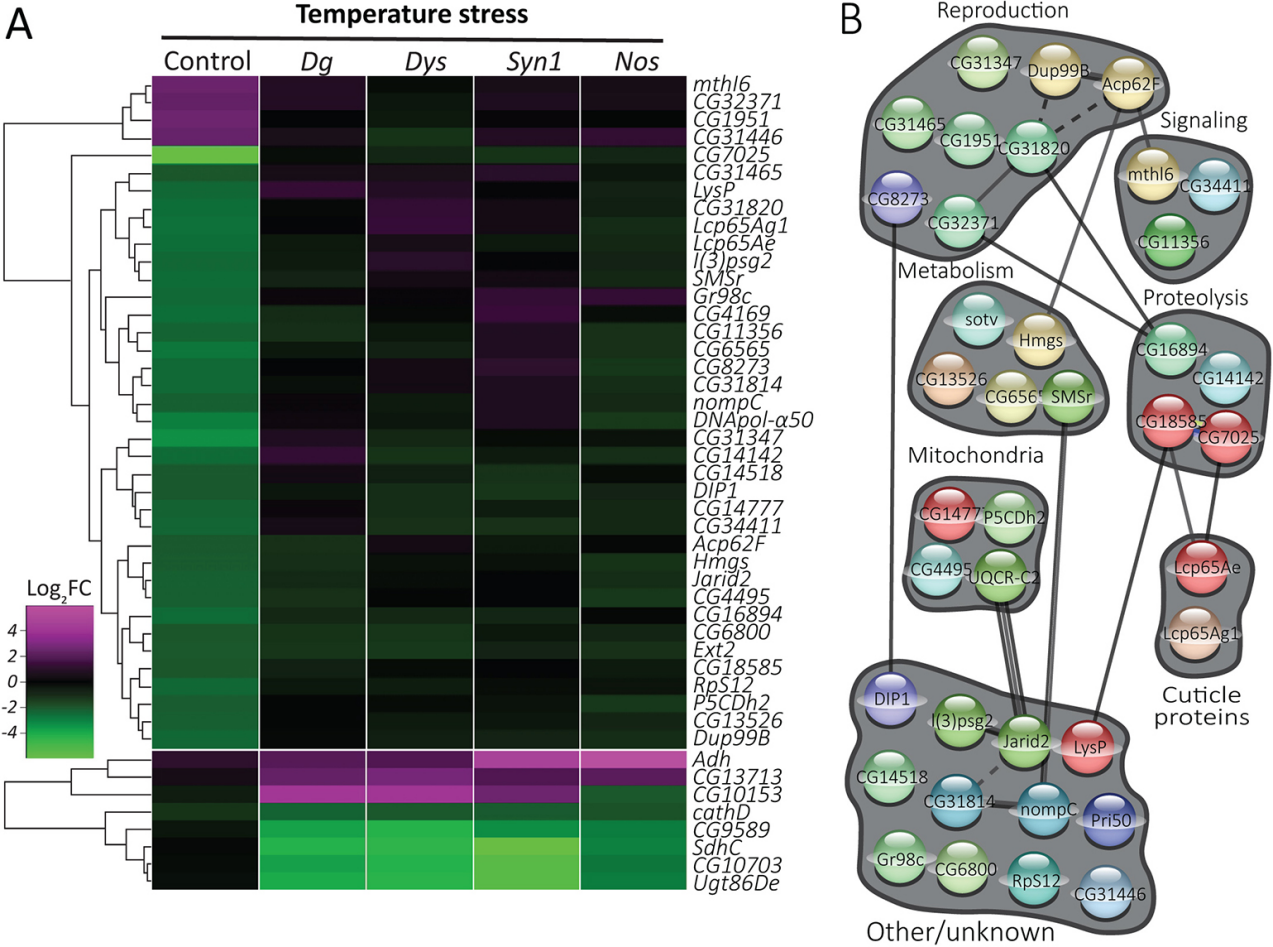
**Defective transcriptional response to temperature stress in DGC mutants.** (A) Heat map depicting DGC-dependent response genes (38 genes; temperature-responsive in control flies, but not in DGC mutants) and DGC-prevented response genes (eight genes; unchanged by temperature in controls, but with altered expression in DGC mutants). (B) STRING-based clustering of the proteins encoded by the DGC-dependent genes depicted in A. See also Fig. S3.

Next, we sought to identify genes and pathways that do not respond appropriately to stress in the absence of a functional DGC. We began by examining our temperature-stress dataset for genes that exhibit a different response to temperature stress in control flies than in DGC mutants. First, we sought genes of which the expression levels change in controls but do not change in any of the DGC mutants. For this purpose, we looked for genes that have an expression change of at least twofold in response to temperature stress in controls, and we considered genes to exhibit no change if their fold change was less than 1.62 (log_2_FC<0.7). This analysis identified 38 genes that did not show altered expression levels in DGC mutants under temperature stress, even though their expression levels changed in controls (Fig. 3A; Table S7). We refer to this class of genes as ‘DGC-dependent response’ genes because the regular changes in their transcription in response to stress depend on an intact DGC. Complementarily, we also sorted for genes that did not exhibit a change in expression levels in control (FC<1.62) but that show altered expression levels of at least twofold in all DGC mutants. We found eight genes with these patterns of expression in response to temperature, which we refer to as ‘DGC-prevented response’ genes as the irregular changes in their expression (i.e. those that are not observed in control flies) are specifically prevented by the normal function of the DGC (Fig. 3A; Table S7).

As it has been shown that MD patients have difficulty maintaining their body temperature (Hayes et al., 2008), this catalog of genes that do not respond appropriately to temperature might provide insight into the biological basis of this phenotype. In both the DGC-dependent response and DGC-prevented response groups, dysregulated metabolic genes emerge (Table S7), indicating that DGC deficiency is detrimental to proper metabolism during stress, both by causing upregulation and downregulation of genes inappropriately and by failing to regulate genes that should be differentially expressed under stress.

Importantly, *mdx* mice, a genetic model of Duchenne MD, are unable to maintain normal body temperature and have increased energy expenditure (Strakova et al., 2018). It has been speculated that *mdx* mice are not able to consume enough food to meet the metabolic demands of continuous muscle regeneration or that the thermoregulatory set point in the brain is defective in the absence of dystrophin. Similarly, *Drosophila Dg* mutants show abnormal metabolism (Kucherenko et al., 2011; Yatsenko et al., 2014a) and exhibit a cryophilic phenotype caused by increased energy (Takeuchi et al., 2009). This altered thermoregulatory behavior has been linked to the increased mitochondrial oxidative metabolism caused by activation of Ca^2+^ influx (Takeuchi et al., 2009). Loss of Dys leads to abnormal, heat-sensitive muscle contractions that are repressed by mutations in Dg, the binding partner of Dys, and can be rescued by blocking the Ca^2+^ channel (Marrone et al., 2011b). Moreover, *Dys* and *Dg* mutants have antagonistically abnormal cellular levels of reactive oxygen species, suggesting that the DGC has a function in the regulation of muscle cell homeostasis (Kucherenko et al., 2011; Marrone et al., 2011b).

**Fig. 4.**
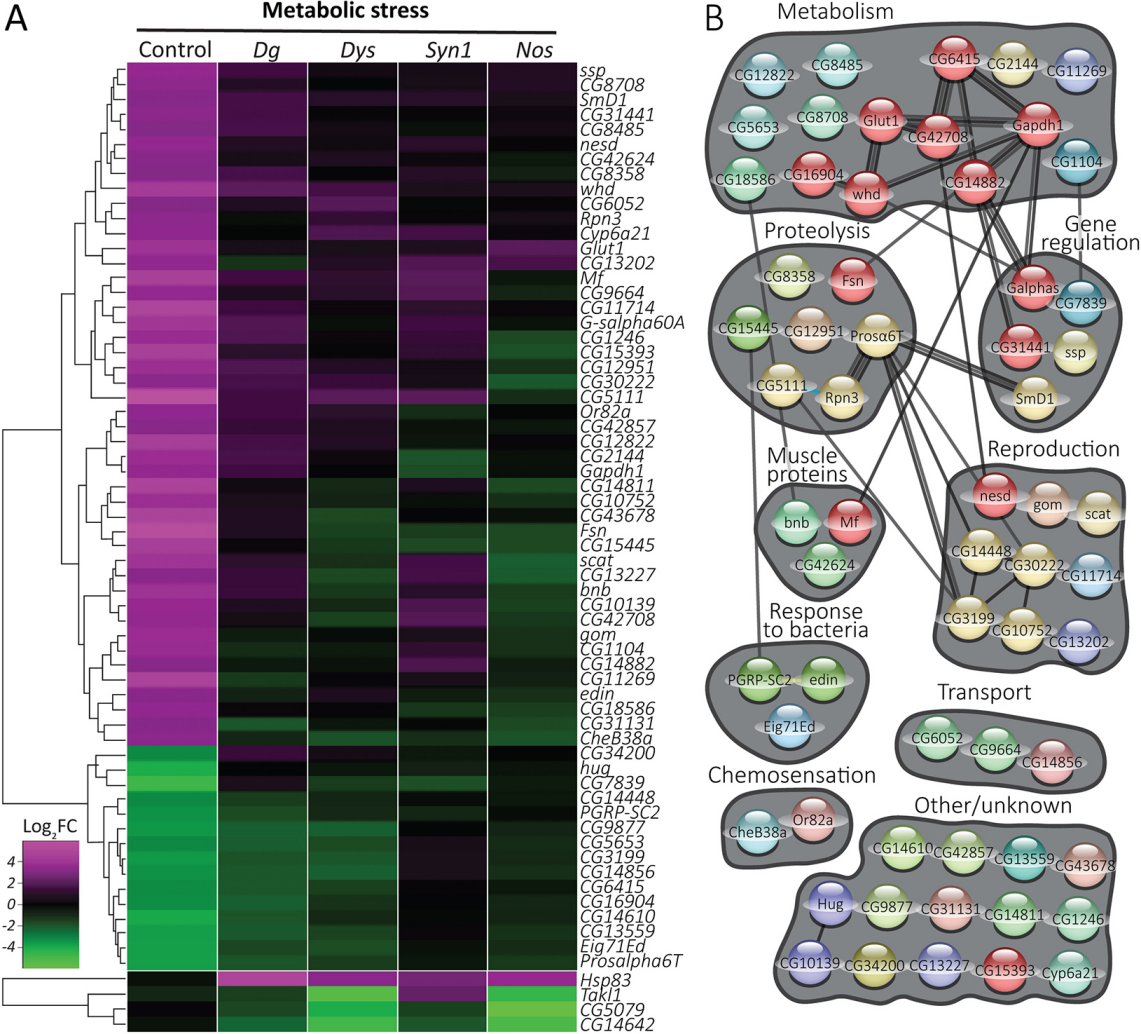
**Defective transcriptional response to metabolic stress in DGC mutants.** (A) Heat map depicting DGC-dependent response genes (61 genes; metabolic stress-responsive in control flies, but not in DGC mutants) and DGC-prevented response genes (four genes; unchanged by metabolic stress in controls, but with altered expression in DGC mutants). (B) STRING-based clustering of the proteins encoded by the DGC-dependent genes depicted in A. See also Fig. S3.

It has been shown previously that dystrophic muscles are already compromised and, as a consequence, they are less adaptive and more sensitive to energetic stress and protein restriction (Kucherenko et al., 2011). Therefore, we were interested in identifying genes that were differentially expressed in the DGC mutants upon dietary restriction. Applying the same analysis to genes dysregulated under metabolic stress, we identified 61 DGC-dependent response genes and four DGC-prevented response genes (Fig. 4A; Table S8). Interestingly, none of the genes with perturbed DGC-dependent expression profiles were common to the temperature- and metabolic stress datasets. This underscores the distinct physiological reactions the organism manifests in response to the different stresses and suggests that a functional DGC is important in mounting both of these responses, albeit through different genetic pathways.

Regarding the metabolic stress ‘DGC-dependent response’ cluster, the most prominent clusters were a group of enzymes involved in metabolism regulation, proteasome proteins and immune response factors (Fig. 4B; Table S8). Among the first group were nutrition-related proteins such as a fatty acid elongase (*CG16904*); a dehydrogenase (glyceraldehyde-3-phosphate dehydrogenase, *Gapdh1*) that binds NAD, regulates the glycolytic process and has biological roles in myoblast fusion, oxidation-reduction, the glucose metabolic process and somatic muscle development; a glucose transporter (*Glut1*); a palmitoyltransferase (*whd*) involved in oxidative stress and metabolic stress; and a GTPase (G protein α s subunit, *Galphas*), which is predicted to enable G-protein-coupled-receptor binding activity and has been shown to positively regulate feeding behavior, response to trehalose and sensory perception of sweet taste (Ueno et al., 2006). Moreover, similarly to *Dys* and *Dg* mutants, *Galphas*-deficient animals exhibit synaptic dysfunctions and have abnormal developmental rates (Hou et al., 2003; Renden and Broadie, 2003).

Interestingly, *whd* mutants exhibit an abnormal immune response (Von Ohlen et al., 2012), and there were other proteins involved in immune response in the DGC-dependent group. Edin is involved in the humoral immune response to Gram-negative bacteria. PGRP-SC2 (Peptidoglycan-recognition protein SC2) is an N-acetylmuramyl-L-alanine amidase that degrades biologically active bacterial peptidoglycans into biologically inactive fragments (Costechareyre et al., 2016; Guo et al., 2014). Recent studies of PGRP-SC2 mutants linked the immune and insulin signaling pathways. It has been shown that PGRP-SC2 downregulation produced insulin receptor (InR)-like phenotypes (Musselman et al., 2018).

Only four genes showed DGC pathway-prevented stress response: *Heat shock protein 83* (*Hsp83*, which encodes the only member of the HSP90 family of chaperone proteins in *Drosophila*) was significantly upregulated, whereas *Tak1-like 1* (*Takl1*), *CG5079* and *CG14642* were downregulated in mutants under metabolic stress but not in controls under the same condition (Fig. 4A).

Heat shock protein 83 (HSP83/HSP90) has been previously associated with response to various stresses in *Drosophila* and in mammals (Biebl and Buchner, 2019; Pearl, 2016). Interestingly, even in the absence of heat stress, the *Hsp83* gene is expressed at high levels in multiple tissues during development (Xiao and Lis, 1989; Zimmerman et al., 1983). HSP90 is a molecular chaperone that promotes the maturation, structural maintenance and proper regulation of specific target proteins involved, for instance, in cell cycle control and signal transduction (Bandura et al., 2013). HSP90 employs the energy of ATP hydrolysis to control the folding and activation of client proteins (Mader et al., 2020). In addition, it dynamically interacts with various co-chaperones that modulate its substrate recognition, ATPase cycle and chaperone function. For example, in muscles, HSP90 binds myosin via a scaffold protein Unc-45 (Ni and Odunuga, 2015). This interaction is required for proper myosin folding and protection from stress (Bujalowski et al., 2018; Lee et al., 2011). Interestingly, in the presence of dietary amino acids, HSP90 is both necessary and sufficient for neuronal stem cell reactivation by promoting the activation of the InR pathway in the developing brain (Huang and Wang, 2018).

In the DGC-dependent groups for both metabolic and temperature stress, there are prominent clusters of metabolic, proteolysis-related and reproduction-related (primarily testis-expressed) genes. Most of the genes in these groups follow the general trend in which genes are downregulated upon temperature stress and upregulated under metabolic stress. For example, 100% of the DGC-dependent metabolic genes [*Ext2* (or *sotv*), *CG6565*, *Hmgs*, *SMSr* and *CG13526*] and the proteolysis-related genes (*CG16894*, *CG18585*, *CG7025* and *CG14142*) in the temperature stress group are downregulated in controls, whereas in the metabolic stress condition, most (75%) of the metabolic genes [*CG1104* (or *Ufl1*), *CG14882*, *CG18586*, *CG42708* (*GLS*), *Gapdh1* and *Glut1*] and 80% of the proteolysis genes (*CG15445*, *CG5111*, *Fsn* and *Rpn3*) are upregulated.

The DGC-dependent temperature- and metabolic stress-dependent genes that are involved in reproduction (Fig. 3B; Fig. 4B), along with those reproduction-related genes that are DGC dependent even in unstressed conditions (Fig. 1D), could present important new insight into the molecular causes of fertility deficits in DGC-deficient mice and flies. In addition to these similarities, the DGC-dependent groups also differ in important ways between temperature and dietary restriction. In temperature stress, there is a group of mitochondrial genes that are downregulated; no such group appears under metabolic stress. Similarly, upon dietary restriction, there is a group of regulatory genes (encoding G proteins, transcription factors and splicing factors), mostly upregulated in controls, whereas no such group appears in temperature stress.

### A subset of genes exhibits DGC-independent responses to temperature and metabolic stress

Interestingly, while generating the DGC-dependent and DGC-prevented response gene datasets, we found almost no genes that are regulated in response to stress in an entirely opposite way in control versus DGC mutants (i.e. upregulated in control but downregulated in all the mutants, or vice versa). Indeed, when we filtered the temperature and metabolic stress datasets such that we could examine all genes that are similarly regulated in the four DGC mutants, we found that there are three categories of genes: (1) those that were changed in controls but not in mutants (DGC-dependent stress response genes); (2) those that were unchanged in controls but showed altered expression in all mutants (DGC-prevented stress response genes); and (3) those that were changed in controls and in DGC mutants. Nearly all of the genes in the latter category were upregulated or downregulated together in all genotypes, including controls (Fig. S3). Overall, these genes respond to temperature stress irrespective of genotype; therefore, we consider them to represent ‘DGC-independent response’ genes.

In the cluster of genes that were changed in all genotypes in response to temperature stress, 90% of genes (19 of 21) were regulated in the same direction (upregulated or downregulated) (Fig. S3A, Table S9). That feature was not a criterion for inclusion in our analysis; rather, we required that a gene was twofold dysregulated in all genotypes, but any gene could have been upregulated in some genotypes and downregulated in others, and still have been included here. But almost all genes were dysregulated in the same direction in all genotypes. The two exceptions were the outliers *bigmax* and *Brd*. Each was downregulated by temperature in all but one genotype: *bigmax* was upregulated in the *Dys* mutant and *Brd* was upregulated in the *Dg* mutant.

Similarly, in the metabolic stress cluster, 88% of genes (37 of 42) followed this pattern, corresponding to a DGC-independent response (Fig. S3B, Table S10). Of the few outliers, most exhibited differential regulation between the DGC mutants. The sole exception was *sro*, which, in response to metabolic stress, was upregulated in control and downregulated in all of the DGC mutants (Fig. S3B, Table S10).

Several lines of evidence in flies and mammals have established the DGC as a signaling hub. First, the association of Nos with Dys establishes the DGC as an important component of the conserved NO signaling pathway. Syn-associated Nos produces NO, which is used in the nitrosylation of intracellular proteins. The nitrosylation of histone deacetylases affects the expression of downstream genes, including those encoding microRNAs, which can have a negative-feedback role on the expression of Dg (Cacchiarelli et al., 2010; Yatsenko et al., 2014b). In addition, the DGC has also been shown to physically associate with Yorkie and Kibra, components of the Hippo signaling pathway, affecting the expression of downstream genes (Morikawa et al., 2017; Yatsenko et al., 2020). Finally, murine Dg (Dag1) and insulin receptor (InR) are closely associated in the muscle sarcolemma. In aging muscle, Dg undergoes increased internalization and degradation via the lysosome, and due to the association of the proteins, InR levels are also reduced. The reduction of InR in aged muscle can be expected to have far-reaching transcriptional effects owing to disruption of the insulin signaling pathway. Indeed, this mechanism might help to explain the insulin insensitivity phenotype exhibited by dystrophic patients (Eid Mutlak et al., 2020). The finding in this study of a cluster of transcriptional regulators whose expression is regulated by the DGC (Fig. 1) implies that the transcriptional effects exerted by the DGC might be more far-reaching than was previously known. Further research will determine whether the transcriptional changes in DGC mutants occur only through these pathways or whether other signaling and transcriptional pathways are involved.

## MATERIALS AND METHODS

### Fly stocks

All fly stocks and crosses were maintained at 25°C on a standard cornmeal-agar-based food in a 12 h/12 h light/dark cycle. Fly stocks used in this study (see also Table S1) were *w^1118^*, *Canton-S*, *w^1118^;Df(2L)BSC230/CyO* and *w^1118^;Df(3L)BSC450/TM6C,Sb^1^cu^1^* from the Bloomington Drosophila Stock Center (BDSC); *Dg^O55^/CyO* and *Dg^O86^/CyO* (kind gifts from Robert Ray, Howard Hughes Medical Institute, Janelia Research Campus); outcrossed *Df(3R)Exel6184* deficiency (Marrone et al., 2012); *Nos^Δ15^* (kind gift from Patrick O'Farrell, University of California, San Francisco); and *ΔSyn1^5-2^/TM3* (see below).

*ΔSyn1* mutants were generated using two transgenic lines, *w^1118^; PBac[WH]CG14565^f05859^* (BDSC #18911) and *P[XP]CG7370^d06092^* (Exelixis Collection, Harvard Medical School) (Thibault et al., 2004), containing transposon elements with FRT sites flanking the *Syn1* gene. In addition, *w^1118^ hsFlp; Dr/TM3,Sb* was used for heat shock-induced Flp recombinase expression. The recombination event was induced by heat shocking embryos and early larvae (0-36 h collection) with the genotype *w^1118^ hsFlp; PBac[WH]CG14565^f05859^/P[XP]CG7370^d06092^* in a 37°C water bath three times for 1 h each in 12 h intervals. Emerging flies with mosaic eye color were crossed to *w^1118^; Ly/TM3,Sb* and their progeny were selected for the white-eye trait as an indicator of the loss of mini-white gene cassettes from both transgenes, which indicates completion of the recombination event, i.e. the deletion in the *Syn1* locus (Fig. S1A) (Parks et al., 2004). Multiple *ΔSyn1^5-2^* alleles were isolated independently using this procedure and molecularly validated as described below. The isolate *ΔSyn1^5-2^* was used in trans over a deficiency as the *Syn1* LOF mutant in all subsequent experiments in this study.

*ΔSyn1* alleles have a ∼26 kb long deletion in the third chromosome removing the *Syn1* gene, which was confirmed by two PCRs (Fig. S1B,B′) using the HotStarTaq Master Mix (QIAGEN) and the following primers: 5′-CAATCAACATGAAGAGCCAACCCA-3′ (*Syn1*-Exon-5-Fw) and 5′-ACTTTGCCGCCGATGTCACTGT-3′ (*Syn1*-Exon-5-Rv) to confirm the absence of the *Syn1* gene (Fig. S1B, red arrows); and 5′-AATGATTCGCAGTGGAAGGCT-3′ (XP5′ plus) and 5′-GACGCATGATTATCTTTTACGTGAC-3′ (WH5′ minus) (Parks et al., 2004) to detect the recombination-resulted junction of the two residual transgene fragments (Fig. S1B′, red arrows). In addition, an absence of *Syn1* mRNA expression in *ΔSyn1* mutants was confirmed by quantitative PCR using High Capacity Reverse Transcriptase (Applied Biosystems) and Fast SYBR Green reagents in a StepOne Plus Real Time PCR System (Applied Biosystems) using the following primers for *Syn1* and *RpL32*: 5′-CCCTCGTCTGGTTCAATGCC-3′ (*Syn1*-Fw), 5′-AATCTCAAATACATCGACCC-3′ (*Syn1*-Rv), 5′-AAGATGACCATCCGCCCAGC-3′ (*RpL32*-Fw) and 5′-GTCGATACCCTTGGGCTTGC-3′ (*RpL32*-Rv). For the calculation of relative mRNA expression, C_T_ values of the housekeeping gene *RpL32* were subtracted from the C_T_ values of *Syn1* to calculate the ΔC_T_ values. The *Syn1* expression in *Oregon-R-C* flies was analyzed as control and for normalization, hence calculation of the ΔΔC_T_ values. Relative expression was calculated with the formula 2^−ΔΔCT^ (Fig. S1C).

### RNA sequencing and data analysis

Samples were prepared for RNA sequencing as follows. One-week-old flies were used with the following genotypes: *Canton S/w^1118^* (control), *Dg^086^/Dg^055^* (*Dg* LOF), *Df(3R)Exel6184/Df(3R)Exel6184* (*Dys* LOF), *Df(3L)BSC450/ΔSyn1^5-2^* (*Syn1* LOF) and *Df(2L)BSC230/Nos^Δ15^* (*Nos* LOF). Flies were kept on standard food for 5 days at 25°C in a 12 h/12 h light/dark cycle for the unstressed condition. In order to induce heat stress, flies were kept in an incubator at 33°C on standard food for 5 days. Metabolic stress was induced by feeding the flies on yeast paste only (yeast paste made from dry yeast mixed with 5% propionic acid in water) at 25°C for 4 days.

For RNA extraction, ∼10 male flies per genotype and condition were homogenized together in Trizol (Ambion) and RNA was extracted using the Direct-zol RNA mini-prep kit with an additional on-column DNAse digestion step (Zymo Research). The quality of the purified RNA was assessed with a Nanodrop ND-1000 spectrophotometer measuring the A260/A280 and A260/A230 ratios. Only RNA with A260/A280>2.0 and A260/A230>1.7 was used for further RNA-seq application. For each sample, 5-10 µg of total RNA was sent to GATC Biotech (Konstanz, Germany) for library preparation and subsequent transcriptome sequencing. In summary, RNA-seq libraries were prepared by RNA poly-A purification, fragmentation, random-primed cDNA synthesis, linker ligation and PCR enrichment. The samples were used to make a random-primed cDNA library, and the run was performed on an Illumina HiSeq platform with single-end, 100 bp reads.

The transcriptome sequencing experiment resulted in a sample average of ∼7.5 million reads that could be mapped to a unique transcript. Genome index was generated from genome FASTA files of individual chromosomes (BDGP6 version) and the transcript annotation GTF file Drosophila_melanogaster.BDGP6.84 (ensemble.org database; Yates et al., 2016). Subsequently, the reads were mapped to the reference genome using STAR: ultrafast universal RNA-seq aligner (Dobin et al., 2013). Subsequent analyses were performed in R statistical software (https://www.r-project.org/) using packages from the Bioconductor project (Gentleman et al., 2004). The resulting BAM alignment files were used to generate counts on individual transcripts using the transcript database from the same GTF file [via the Rsamtools (https://bioconductor.org/packages/Rsamtools) and GenomicFeatures (Lawrence et al., 2013) packages] and the ‘summarizeOverlaps’ function in ‘Union’ mode via GenomicAlignments package (Lawrence et al., 2013). In total, 15,930 unique transcripts were detected with at least one count. Next, the counts were analyzed and the genotype and condition comparisons were done through built-in statistical models in the DESeq2 package (Love et al., 2014). For data visualization, gplots (https://cran.r-project.org/package=gplots) and RColorBrewer (Neuwirth, 2011) packages were used.

For differential gene expression analysis, twofold difference and *P*-values smaller than 0.1 were considered significant and filtered by the ‘results’ function with ‘lfcthreshold=1’ and subsetting the genes with *P*<0.1. The resulting gene lists were subjected to gene interaction and ontology term analysis using STRING and DAVID databases, and ClueGO (Bindea et al., 2009) and CluePedia applications via Cytoscape software (Shannon et al., 2003).

## Supplementary Material

10.1242/dmm.049862_sup1 Supplementary information
