# Supplementary material for: Signaling through the dystrophin glycoprotein complex affects the stress-dependent transcriptome in Drosophila
Source: Dis Model Mech. 2023 Jan 31;16(1):dmm049862. doi: 10.1242/dmm.049862 (PMC9922874; doi:10.1242/dmm.049862)
Supplement: Supplementary information [file dmm-16-049862-s1.pdf]

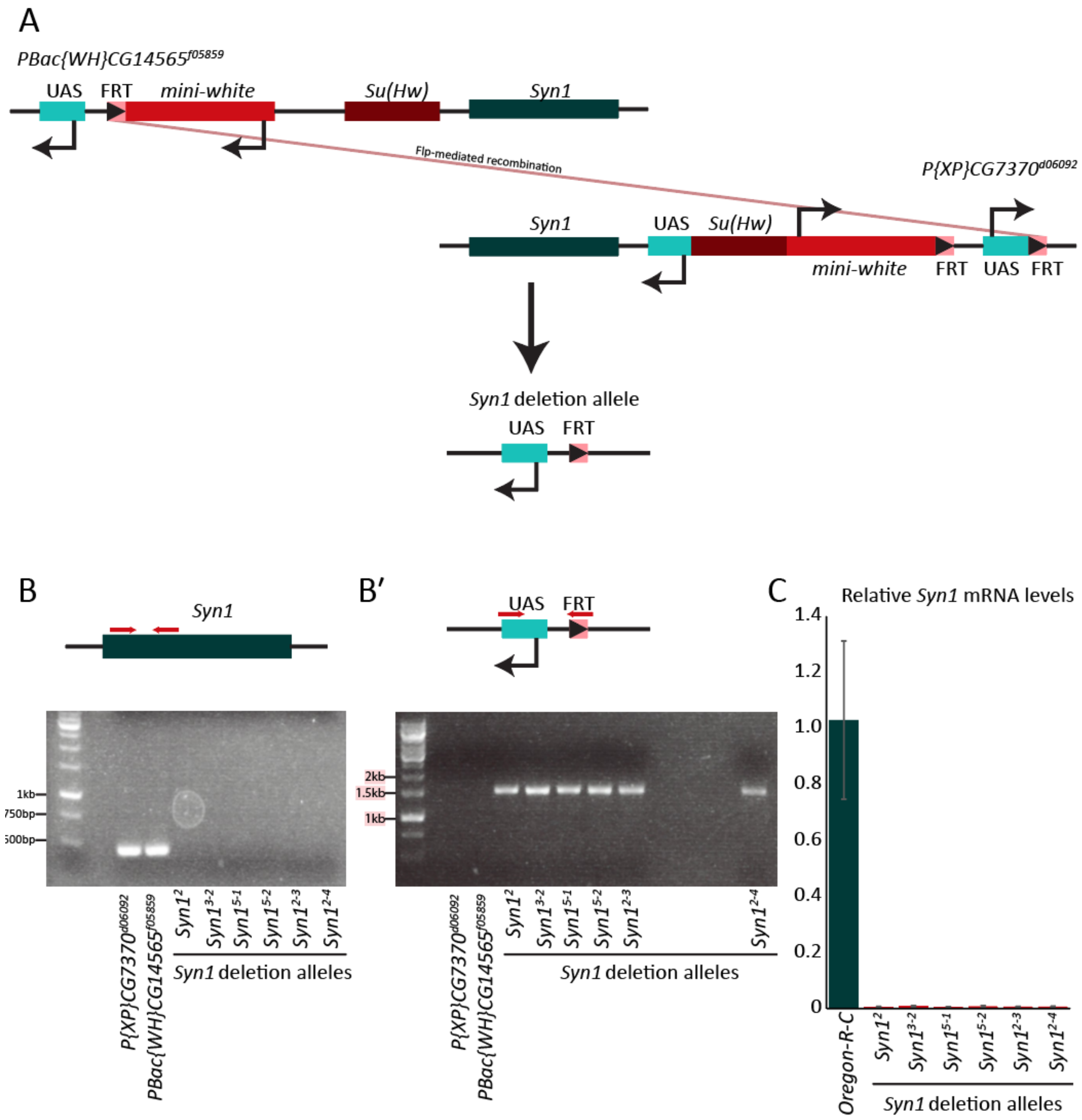

Fig. S1. Generation of new *Syntrophin1* deficiency, *ΔSyn1*

A. Deficiency was generated using Flp-mediated recombination between two FRT-containing transposons flanking the *Syn1* gene (*PBac[WH]CG14565^{f05859}* and *P[XP]CG7370^{d06092}*; see Materials and Methods).

B-B′. PCR confirmation of new *ΔSyn1* alleles. (B) Primers specific for the endogenous *Syn1* gene result in PCR amplification in the parental strains *PBac[WH]CG14565^{f05859}* and *P[XP]CG7370^{d06092}*, but no bands are present in lanes representing *Syn1* deletions. (B′) Primers that recognize residual transposon fragments flanking the new deficiency positively detect the presence of the deficiency in flies with deletion alleles, but not in parental flies.

C. RT-qPCR confirms the absence of RNA from *Syn1* deletion mutants, while it is readily detected in control flies (Oregon-R-C).

Disease Models & Mechanisms • Supplementary information

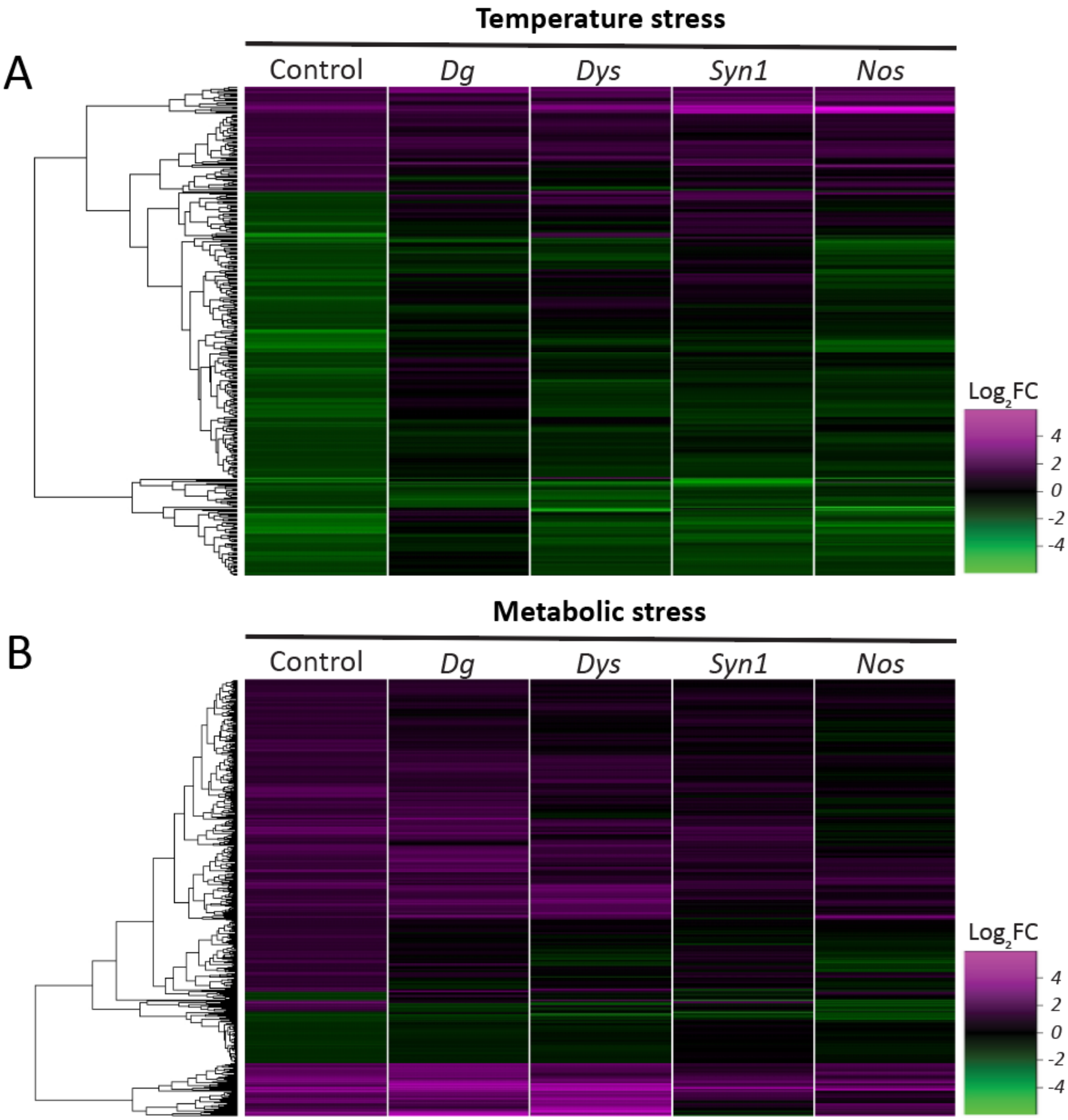

Fig. S2. All genes dysregulated by temperature and metabolic stress (related to Figure 2)

A-B. Heat maps with all genes dysregulated in control flies by temperature stress (A; 357 genes) and metabolic stress (B; 483 genes), also showing the expression level of these genes in the four DGC mutants.

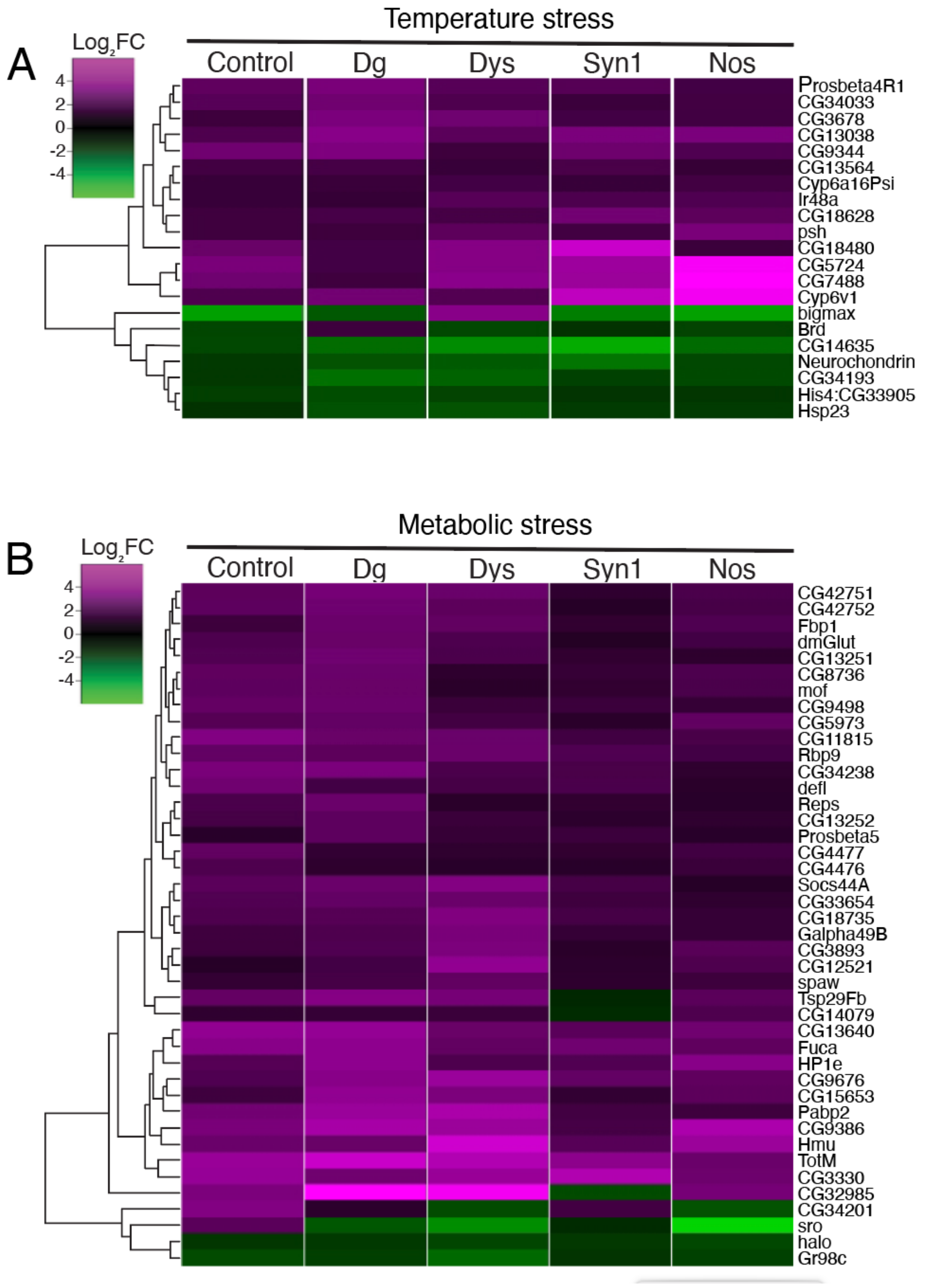

Fig. S3. DGC-independent genes are regulated by stress irrespective of presence of a functional DGC (related to Figures 3 and 4)

A-B. Heat maps showing all genes that are dysregulated by temperature stress (A; 21 genes) or metabolic stress (B; 42 genes) in all genotypes, including controls, indicating a DGC-independent stress-response mechanism. Of these genes, 90% of temperature-responsive genes and 88% of metabolic-responsive genes are dysregulated similarly (up- or downregulated) in all genotypes.

Disease Models & Mechanisms • Supplementary information

Table S1. Log2-FC values for all comparisons

Click here to download Table S1

Table S2. 46 genes dysregulated in all 4 DGC mutants: Dg Dys Syn1 & Nos

Click here to download Table S2

Table S3. 71 genes dysregulated in 3 mutant genotypes: Dg, Dys, & Syn1 (when Nos is excluded)

Click here to download Table S3

Table S4. 357 genes dysregulated by temperature stress (77 upregulated and 280 downregulated)

Click here to download Table S4

Table S5. 483 genes dysregulated by metabolic stress (416 upregulated and 67 downregulated)

Click here to download Table S5

Table S6. 59 genes dysregulated by both temperature stress and metabolic stress

Click here to download Table S6

Table S7. DGC-dependent and DGC-prevented temperature-response genes

Click here to download Table S7

Table S8. DGC-dependent and DGC-prevented metabolic-response genes

Click here to download Table S8

Table S9. DGC-independent temperature-response genes

Click here to download Table S9

Table S10. DGC-independent metabolic stress-response genes

Click here to download Table S10
